# Neuroprotective effect of HLDF-6-H peptide in Parkinson’s disease

**DOI:** 10.1192/j.eurpsy.2025.1705

**Published:** 2025-08-26

**Authors:** Y. Zolotarev, O. Dolotov, D. Markov, S. Shram, A. Dadayan, N. Kost, O. Sokolov, S. Zozulya, E. Shubina

**Affiliations:** 1 NRC «Kurchatov Institute»; 2 Lomonosov Moscow State University, Faculty of Biology; 3 FSBSI “Mental Health Research Center”; 4 All-Russian Charitable Foundation “Movement - Life”, Moscow, Russian Federation

## Abstract

**Introduction:**

The pathogenesis of Parkinson’s disease (PD) is associated with simultaneous damage to the nervous, endocrine and immune systems. Therefore, drugs that have a regulatory effect on all of these systems should be used to treat PD. Similar properties are possessed by the synthetic analogue of fragment 41–46 Human Leukemia Differentiation Factor (Thr-Gly-Glu-Hse-His-Arg-NH2, HLDF-6-H)

**Objectives:**

To study the effects of HLDF-6-H in an experimental model of PD and its impact on the severity of motor and non-motor symptoms in patients with PD

**Methods:**

The study used a preclinical PD model based on the administration of moderate doses of MPTP toxin (18 mg/kg) and chronic intranasal administration of HLDF-6-H peptide (300 μg/kg) to C57Bl/6 mice. Mice behaviour was assessed in the Grid test and the Forced Swim (FS) test. After 3 weeks of peptide administration, mRNA of neurotropic factors (BDNF and NGF) and key cytokines (IL-1β, IL-6, IL-10, gamma interferon, tumour necrosis factor α and transforming growth factor β1) was analyzed in five brain regions (striatum, hippocampus, hypothalamus, pituitary gland, cortex). Serum levels of 10 steroids, including testosterone, estradiol, progesterone, and corticosterone, were determined using MS analysis. The activity of inflammatory markers leukocyte elastase (LE) and α1-proteinase inhibitor (α1-PI) was determined using kinetic methods. Patients (24 people) with a disease duration from 1 to 16 years received HLDF-6-H intranasally as part of the balm “Rinohealing” (9-31 μg/kg per day, up to 6 months) in addition to standard pharmacotherapy for PD. The effectiveness of therapy was assessed based on the patients’ subjective assessment of their condition according to the MDS-Unified Parkinson’s Disease Rating Scale (MDS-UPDRS)

**Results:**

HLDF-6-H blocked motor disorders (Grid test) and depressive-like syndrome (FS test) caused by MPTP in mice, restored the level of neurotropic factors and cytokines in the brain of animals (p<0.05). In the model used, a decrease in the level of estradiol and cortisol in the blood was reversible by the peptide (p<0.05). The development of the inflammatory process under MPTP action is indicated by an increase in the activity of α1-PI, the anti-inflammatory effect of HLDF-6-H is expressed in a decrease in the serum activity of LE and α1-PI (p < 0.05). A decrease in the severity of a number of motor (rigidity, tremor, movement and balance disorders) and non-motor (anxiety-depressive state, impairment of memory, cognitive functions, sleep, etc.) pathological symptoms of PD was noted with chronic use of “Rinohealing” as an additional therapy.

**Conclusions:**

HLDF-6-H peptide has antidepressant, neuroprotective and anti-inflammatory activity. This has been demonstrated both in the experimental model of PD and when used as an additional therapy for PD. The obtained results indicate the prospects for further studies of HLDF-6-H for the treatment of PD.

**Disclosure of Interest:**

None Declared

